# Rapid functional cardiac imaging after gadolinium injection: Evaluation of a highly accelerated sequence with sparse data sampling and iterative reconstruction

**DOI:** 10.1038/srep38236

**Published:** 2016-12-01

**Authors:** Johannes Budjan, Holger Haubenreisser, Thomas Henzler, Sonja Sudarski, Michaela Schmidt, Christina Doesch, Ibrahim Akin, Martin Borggrefe, Nadja M. Meßner, Stefan O. Schoenberg, Ulrike I. Attenberger, Theano Papavassiliu

**Affiliations:** 1 Department of Clinical Radiology and Nuclear Medicine, University Medical Center Mannheim, Medical Faculty Mannheim, Heidelberg University, Germany; 2 DZHK (German Centre for Cardiovascular Research) partner site Mannheim, Germany; 3 MR Product Innovation and Definition, Siemens Healthcare GmbH, Erlangen, Germany; 4 1st Department of Medicine, University Medical Center Mannheim, Medical Faculty Mannheim, Heidelberg University, Germany; 5 Computer Assisted Clinical Medicine, University Medical Center Mannheim, Heidelberg University, Mannheim, Germany

## Abstract

To generate a patient-friendly, time-efficient cardiac MRI examination protocol, a highly accelerated real-time CINE MR sequence (SSIR) was acquired in the idle time in between contrast injection and late gadolinium enhancement phase. 20 consecutive patients underwent a cardiac MRI examination including a multi-breath-hold sequence as gold standard (Ref) as well as SSIR sequences with (SSIR-BH) and without breath-hold (SSIR-nonBH). SSIR sequences were acquired 4 minutes after gadolinium injection. Right- (RV) and left-ventricular (LV) volumetric functional parameters were evaluated and compared between Ref and SSIR sequences. Despite reduced contrast between myocardium and intra-ventricular blood, volumetric as well as regional wall movement assessment revealed high agreement between both SSIR sequences and Ref. Excellent correlation and narrow limits of agreements were found for both SSIR-BH and SSIR-nonBH when compared to Ref for both LV (mean LV ejection fraction [EF] Ref: 52.8 ± 12.6%, SSIR-BH 52.3 ± 12.9%, SSIR-nonBH 52.5 ± 12.6%) and RV (mean RV EF Ref: 52.7 ± 9.4%, SSIR-BH 52.0 ± 8.1%, SSIR-nonBH 52.2 ± 9.3%) analyses. Even when acquired in the idle time in between gadolinium injection and LGE acquisition, the highly accelerated SSIR sequence delivers accurate volumetric and regional wall movement information. It thus seems ideal for very time-efficient and robust cardiac MR imaging protocols.

Cardiac magnetic resonance imaging (CMR) is widely accepted as the standard of reference for the non-invasive assessment of cardiac function. With CMR, the change of both right- (RV) and left-ventricular (LV) volume can be measured throughout the cardiac cycle. LV and RV ejection fractions (EF), basic surrogate parameters of cardiac function, can be derived from those volumetric assessments. As the degree of EF reduction and its development over time provide both information about the severity of the individual patient’s disease as well as the prognosis, this value can influence decision making in the therapy of various acute and chronic, congenital and acquired cardiac pathologies^1^,^2^. Exact LV and RV volumetric assessment thus is crucial in different clinical settings. Above that, functional CMR allows the assessment of regional LV and RV wall movement as well as the calculation of LV mass.

In clinical routine, a stack of short-axis views covering the entire LV and RV is acquired using ECG-gated segmented cine sequences for the volumetric and wall movement assessments^3^. As conventional cine sequences require a breath-hold for each short-axis view, acquisition times for a stack of short axis views covering the entire heart range between 3–4 minutes. Especially when acquisition is performed in expiration, patients need certain time to recover from the previous breath-hold, until the next short axis view can be acquired. Depending on the individual patient’s capabilities, the acquisition of all cine views (including long-axis views) will take roughly 10 minutes, the short axis stack alone requiring between 5 and 7 minutes.

Besides the volumetric parameters, CMR provides information about myocardial fibrosis by using late gadolinium enhancement (LGE) imaging^4^,^5^,^6^. LGE is typically acquired starting 7–10 min after the administration of gadolinium-based contrast agents (GBCA)^3^. As GBCAs influence the contrast between myocardium and blood pool and thus can impair delineation of the ventricular myocardium borders, sequences used for volumetric assessment are typically acquired as a separate protocol module prior GBCA admission^3^. This leads to a substantial idle time between contrast injection and the start of LGE acquisition. An approach to reduce scanning time is to inject GBCA right at the start of the examination, acquire cine sequences during the waiting time for LGE and finish with LGE acquisition. This however results in an imaging protocol with a high frequency of breath-hold commands and little time for patients to recover between the different protocol steps. Especially in patients with severely impaired cardiac function, breath-hold capabilities typically decline after some time, and image quality will likely be more and more reduced by motion artifacts throughout the examination.

Various new techniques have been developed to reduce acquisition time and allow artifact-free imaging even in severely ill patients with reduced breath-hold capabilities, of which compressed sensing techniques are considered to offer a highly promising approach in this field^7^,^8^,^9^,^10^. A sequence using sparse data sampling and non-linear iterative image reconstruction (SSIR) with k-t regularization was recently shown to allow an ultra-fast acquisition of a stack of short-axis views during a single breath-hold or even during free breathing. This sequence was shown to provide exact volumetric parameters when compared to a multi-breath-hold reference sequence for both LV^10^ and RV^9^.

Aim of this study was to evaluate the accuracy of a comparable SSIR sequence for quantitative and qualitative right- and left-ventricular assessment acquired after GBCA admission in the idle time prior LGE acquisition. This was done in order to create a lean, time-efficient imaging protocol including both sequences for volumetric as well as LGE assessment.

## Results

**Figure 1:**
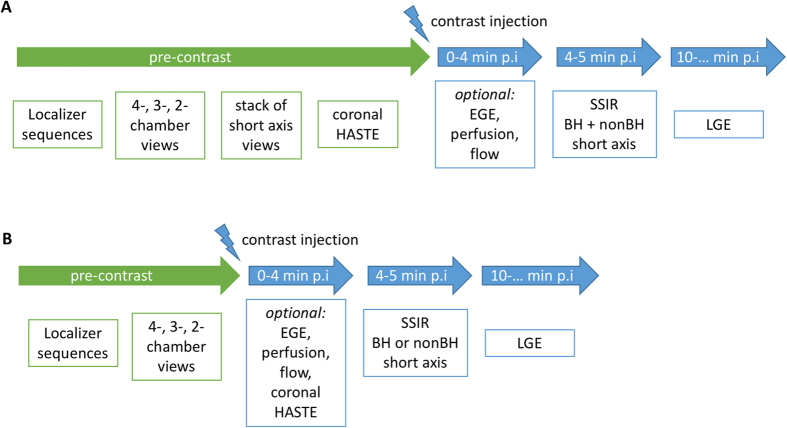
Sequence protocol overview for (**A**) study and (**B**) example of a time-efficient protocol using SSIR. HASTE: half-Fourier single-shot turbo spin-echo sequence, EGE: early gadolinium enhancement, BH: breath-hold, LGE: late gadolinium enhancement.

**Figure 2:**
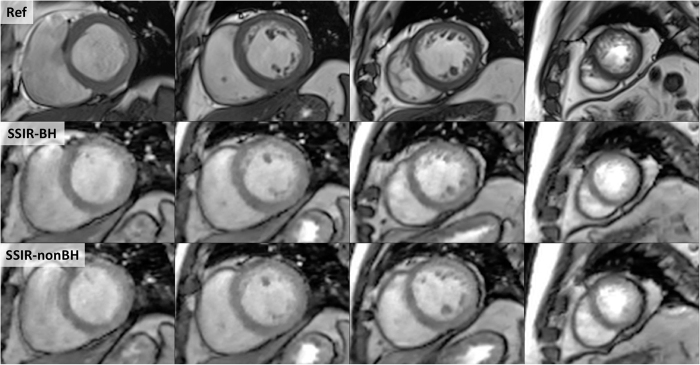
Direct comparison of reference sequence (Ref, top row) and SSIR sequence with (SSIR-BH, middle row) and without (SSIR-nonBH, bottom row) breath-hold. 4 exemplary short-axis views from basal to apical (left to right).

**Figure 3:**
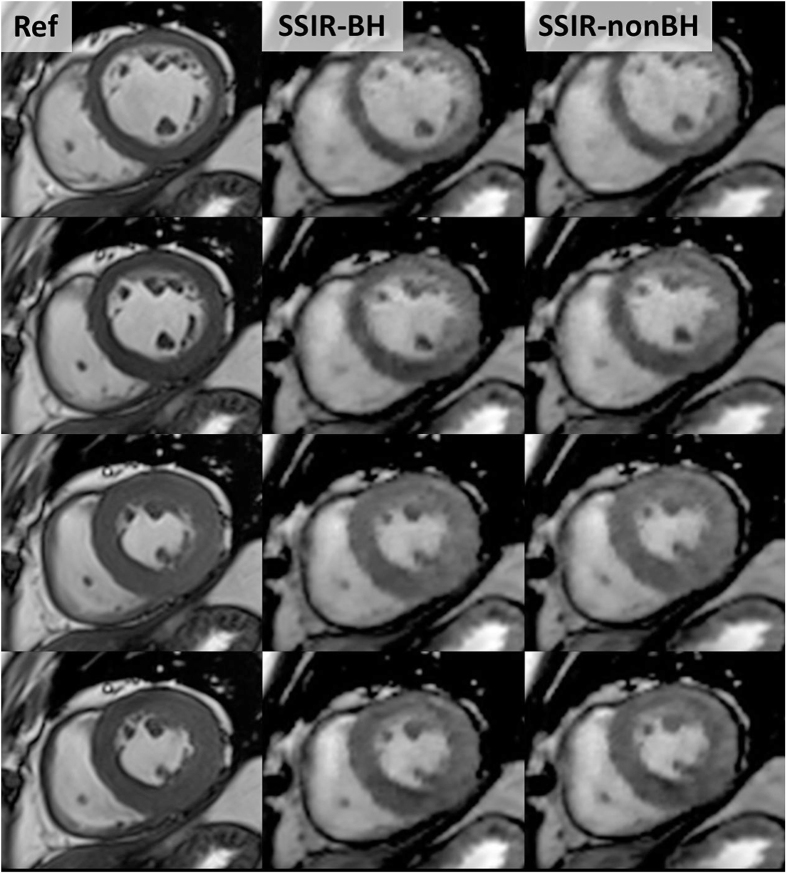
Direct comparison of reference sequence (Ref, left column) and SSIR sequence with (SSIR-BH, middle column) and without (SSIR-nonBH, right column) breath-hold. 4 exemplary short-axis views from end-systolic to end-diastolic phase (top to bottom).

In all 20 patients, Ref, SSIR-BH and SSIR-nonBH sequences were acquired successfully according to the imaging protocol (Fig. 1A). The patients were referred to the CMR examination with known or suspected ischemic (n = 4), dilatative (n = 4), hypertrophic (n = 3), non-compaction (n = 1), arrhythmogenic right-ventricular cardiomyopathy (n = 1), cardiac involvement in sarcoidosis (n = 1) and amyloidosis (n = 1) and myocarditis (n = 5). All datasets were successfully loaded to the offline tool and manual contouring was possible for both RV and LV in all datasets. Delineation of both endocardial and epicardial border for LV and endocardial border for RV was possible from basis to apex (Fig. 2) throughout the cardiac cycle from diastole to systole (Fig. 3).

Tables 1 and 2 give an overview over the results of the volumetric assessment. For the LV measurements, excellent correlation and narrow limits of agreements were found for both SSIR-BH and SSIR-nonBH when compared to Ref. Even though LVEDV for both SSIR sequences were found to be statistically significant different compared to Ref, Bland-Altman analysis revealed only a slight underestimation of those values. Using SSIR sequences, measurements of LVESV and LVEF volumes showed no statistically significant difference compared to Ref. Using SSIR-nonBH, LVMass was slightly underestimated. In the RV volumetric assessments, both SSIR sequences showed no statistically significant differences compared to Ref. In the intra- and inter-observer variability assessment, high correlation between the evaluations was found for LVEF and RVEF in both SSIR sequences (Table 3).

**Figure 4:**
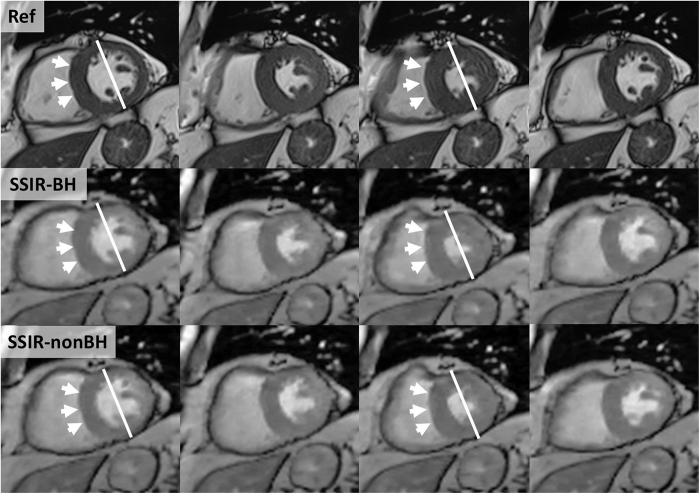
Patient with relative hypokinesia of the septal segments (arrows). Direct comparison of reference sequence (Ref, top row) and SSIR sequence with (SSIR-BH, middle row) and without (SSIR-nonBH, bottom row) breath-hold. The thickening of lateral wall segments during systole is clearly visible in all three sequences.

In the visual RMW assessment of the 320 LV segments, 6 segments were rated as dyskinetic and 44 as hypokinetic using Ref. 5 segments (1,5%) that were rated hypokinetic in Ref were incorrectly rated as akinetic using both SSIR sequences, 1 segment was incorrectly rated hypokinetic in SSIR-BH (normal in Ref and SSIR-nonBH). 314 segments (98%) received identical ratings in all three sequences (Fig. 4). Overall, an excellent agreement between both Ref and SSIR-BH (ĸ = 0.94) as well as Ref and SSIR-nonBH (ĸ = 0.94) was found. Similar results were found in the evaluation of the intra- and inter-observer agreement, which was excellent for both SSIR-BH and SSIR-nonBH (Table 3). In the assessment of RMW for the RV, all 100 segments received identical ratings in all three sequences and all readings (15 hypokinetic, no dys- or akinetic segments).

## Discussion

Our findings demonstrate that a SSIR sequence can be used to acquire short-axis datasets for LV and RV volumetric analysis in the idle time between gadolinium injection and LGE acquisition. Despite the reduced contrast between myocardium and intra-ventricular blood, both endocardial and epicardial border for LV and endocardial border for RV could be delineated with high accuracy in both SSIR sequences, which thus can be used for volumetric as well as regional wall movement assessment.

For both SSIR sequences, LVEDV was statistically significantly lower compared to Ref. This could be explained partly by the difference of using prospective triggering in the SSIR sequences, which leads to missing of the very late diastole. In contrast, Ref uses retrospective gating and thus covers the full cycle. However, with a difference of about 3 ml, this variance can be considered not clinically relevant. This is also supported by the LVEF results, which were equal to Ref in both SSIR sequences.

In order to create a time-efficient protocol, gadolinium-based contrast agents can be injected at the start of the examination when using conventional cine techniques. Such protocols are widely used in clinical practice. In our opinion, using a highly accelerated technique in this setting provides four distinct advantages. First, using conventional sequences, patients will have to follow consecutive breath-hold commands throughout the entire examination. Depending on the patients’ overall condition, their ability to follow entire breath-hold intervals typically decreases over the course of the examination. This is especially true as such time-optimized protocols typically cannot include sufficiently long recreational phases. Using the approach proposed in our study (Fig. 1B), patients can recover for up to 4 minutes prior short-axis stack acquisition, followed by another recreation time of 3–5 minutes until LGE acquisition starts. We expect these relatively long intervals to have a positive impact on patients’ comfort and compliance and consequently on image quality of LGE images. Second, using the proposed protocol, LGE acquisition can be performed at a very standardized time after gadolinium injection and the overall protocol can be acquired in a very standardized order. Using conventional cine sequences, the interval prior to LGE acquisition is packed with continuous sequence acquisition. Problems during the acquisition, such as strong breathing artifacts due to temporary patient incompliance in a certain step and its repetition might lead to a prolonged examination time. This consequently can lead to a shift of LGE acquisition time points, resulting in varying contrast of LGE images. In order to facilitate comparison of intra-individual follow-up or inter-individual examinations, our proposed protocol allows enough time to reacquire individual protocol parts while guaranteeing a rather fixed LGE acquisition start time. Third, our proposed protocol can be combined with other optional imaging *modules* such as early enhancement or rest perfusion studies during the first 4 minutes after gadolinium injection. This additional protocol steps would not influence the overall acquisition time. Fourth, in single-dose gadolinium injection protocols, LGE acquisition should start even earlier than when using double-dose protocols. This could allow for an even more time-efficient protocol with SSIR short-axis stack acquisition after 4 minutes, directly followed by LGE acquisition. Even though not evaluated in this study, it is very likely that the SSIR technique can also be used in single-dose injection protocols.

Besides critically ill patients, pediatric patients needing sedation for MRI examinations can profit from the proposed protocol. As the SSIR short-axis stack can also be acquired under free breathing, the proposed protocol can be used in sedated patients, minimizing acquisition and subsequently sedation time.

In contrast to previous works on a similar SSIR sequence^9^,^10^, an improved image reconstruction mode was available in this study. This reconstruction mode allows a step-wise background reconstruction of the SSIR datasets during the acquisition of the next sequence steps of the CMR protocol. Thus, SSIR image reconstruction did not directly influence the acquisition in this study. Besides image reconstruction time, the time required for volumetric analysis is of high relevance for clinical routine. In contrast to the fully manual contouring approach performed in this study, automatic contouring software is widely used in clinical routine. We tested two commercially available software packages (syngo Argus 4D VF, Siemens Healthineers, Germany and cvi^42^, Circle Cardiovascular Imaging Inc., Canada) on 10 patients and found that the implemented automatic contouring algorithms worked more efficiently when using the Ref datasets. This lead to a longer time required for the volumetric analysis of SSIR sequences when including semiautomatic volumetric techniques (on average approx. 80 s for Ref vs. 120 s for SSIR-BH and 140 s for SSIR-nonBH for endo- and epicardial contouring in end-diastole and endocardial contouring in end-systole). Especially for the epicardium, the automatically assigned contours had to be corrected considerably in the SSIR datasets. However, with improvements of automatic contouring algorithms, this limitation might be overcome. The relatively low number of patients included has to be considered as the main limitation of this study. Additionally, the patients included in this study showed pathologies mainly affecting LV. The results regarding the SSIR sequence’s robustness in the detection of RV pathologies are thus somewhat limited. Further evaluation in a larger patient cohort, preferably with pathologies involving RV, are necessary to confirm the results found in this study.

In conclusion, the highly accelerated SSIR sequence delivers accurate volumetric and regional wall movement information, even when acquired in the idle time in between gadolinium injection and LGE acquisition. It thus seems ideal for very time-efficient and robust cardiac MR imaging protocols.

## Methods

### Patients

20 consecutive patients (mean age 56 years [range 18–80 years], 6 female) with different suspected or known cardiac pathologies that were planned for late gadolinium enhancement studies were included in this prospective. The institutional review board (Medizinische Ethikkommmision 2, Medizinische Fakultät Mannheim) approved this study and performed in adherence to the Declaration of Helsinki. Written informed consent was obtained.

### MR imaging

All patients were examined with a clinical whole-body 3 T system (Magnetom Skyra, Siemens Healthcare, Erlangen, Germany) using the inbuilt 32-element spine matrix coil and an 18-element body matrix coil. After localizer sequences, standard views including a stack of short-axis views were acquired according to^3^. As a reference standard, a segmented 2D TrueFISP cine sequence with retrospective ECG triggering (Ref) was used. 0.2 mmol/kg body weight Gd-DOTA (Dotarem, Guerbet, France) was injected at a flow rate of 1 ml/s via an antecubital venous access. As part of this study, a prospective triggered real-time 2D TrueFISP cine prototype sequence with incoherent **s**parse data **s**ampling and non-linear, **i**terative SENSE-type reconstruction (SSIR) implementing spatio-temporal regularization using redundant Haar wavelets^11^ was acquired twice 4 minutes after contrast media injection, in a single breath-hold (SSIR-BH) as well as in free shallow breathing (SSIR-nonBH), respectively. The time interval after gadolinium injection of 4 minutes was chosen after a small initial explorative series. Initially, the interval between gadolinium injection and SSIR sequence acquisition was varied from 2 to 7 minutes. Although not evaluated quantitatively, the delineation of the endomyocardial border was found to be sufficient starting 3–4 minutes after injection of gadolinium at double dose (0.2 mmol/kg body weight). At earlier acquisition time points, the reduction of myocardial-blood-pool contrast, probably due to first-pass-perfusion-like effects, was found to be too pronounced for reliable volumetric assessment.

Late gadolinium enhancement sequences were acquired starting 10 min p.i. The examination protocol is shown in Fig. 1, sequence parameter details are given in Table 4.

### Image analysis

All examinations were exported to a dedicated, off-line software (cvi^42^ Version 5.1, Circle Cardiovascular Imaging Inc., Canada) for volumetric and wall motion assessment. The following 9 parameters were assessed for Ref as well as both SSIR-BH and SSIR-nonBH: LV ejection fraction (LVEF), LV end-diastolic volume (LVEDV), LV end-systolic volume (LVESV), LV stroke volume (LVSV), average LV mass (LVMass), RV ejection fraction (RVEF), RV end-diastolic volume (RVEDV), RV end-systolic volume (RVESV) and RV stroke volume (RVSV). Volumetric assessment was performed by a radiologist with 4 years of experience in CMR. Papillary muscle mass was attributed to the LV chamber volume for calculation of LV mass and functional parameters. The end-systolic and end-diastolic phases were picked manually as being the phases with lowest and highest ventricular volumes, respectively. For intra-observer variability assessment, the parameters were measured in 10 randomly selected patients twice. For inter-observer variability assessment, the same 10 patients were evaluated by a second reader with 4 years of experience in CMR.

Regional wall movement (RWM) was assessed using a 16-segment model for LV^12^ and using a 5-segment model for RV by a cardiologist with 10 years of experience in CMR. Wall movement was assessed on a 4-point scale ranging from 1 to 4 (1: dyskinetic, 2: akinetic, 3: hypokinetic, 4: normal). RWM was assessed twice in 10 randomly selected patients for intra-observer variability assessment as well as by a second reader with 4 years of experience in CMR for inter-observer variability assessment.

All readers were blinded to the clinical history of the patients and to whether a SSIR sequence was performed with or without breath-hold. The datasets of the different patients were assessed in a random order.

### Statistical analysis

Statistical analysis was performed using a dedicated software (JMP 11.0, SAS Institute, NC, USA). Shapiro-Wilk test was applied to test for normal distribution of data. The 9 volumetric values of SSIR-BH and SSIR-nonBH were compared to Ref using Bland-Altman and linear regression analysis as well as paired two-sided t-testing. For the inter-observer variability assessment, volumetric values obtained by both readers were compared using linear regression analysis and paired t-testing for the parameters LVEF and RVEF. For RWM analysis, agreement between Ref and both SSIR sequences as well as the intra- and inter-observer agreements were calculated using Cohen’s kappa statistics. Results of Bland-Altman analysis are given as mean difference ((Ref - SSIR)/2), its standard deviation, as well as lower and upper limits of agreement. Linear regression analysis results are given as r^2^ and slope. A p-value of < 0.05 was considered statistically significant.

## Additional Information

**How to cite this article**: Budjan, J. *et al*. Rapid functional cardiac imaging after gadolinium injection: Evaluation of a highly accelerated sequence with sparse data sampling and iterative reconstruction. *Sci. Rep.* **6**, 38236; doi: 10.1038/srep38236 (2016).

**Publisher’s note:** Springer Nature remains neutral with regard to jurisdictional claims in published maps and institutional affiliations.

## Figures and Tables

**Table 1:** Overview of volumetric parameters for reference sequence (Ref) and the study sequences with (SSIR-BH) and without (SSIR-nonBH) breath-hold.

		Mean (±SD)	95% CI	vs. Ref
**Ref**	LVEDV [ml]	200.3 (±81.3)	162.3 to 238.4	—
	LVESV [ml]	101.6 (±65.8)	70.8 to 132.4	—
	LVSV [ml]	98.7 (±24.6)	87.2 to 110.2	—
	LVEF [%]	52.8 (±12.6)	46.9 to 58.7	—
	LVMass [g]	149.3 (±58.4)	121.9 to 176.6	—
	RVEDV [ml]	190.9 (±84.4)	149.0 to 232.9	—
	RVESV [ml]	92.3 (±47.4)	68.8 to 115.9	—
	RVSV [ml]	98.6 (±41.6)	77.9 to 119.3	—
	RVEF [%]	52.7 (±9.4)	48.1 to 57.4	—
**SSIR-BH**	LVEDV [ml]	197.7 (±82.6)	159.0 to 236.3	0.0084^*^
	LVESV [ml]	101.7 (±67.7)	70.0 to 133.3	0.96
	LVSV [ml]	96.0 (±24.1)	84.7 to 107.3	0.0009^*^
	LVEF [%]	52.3 (±12.9)	46.3 to 57.3	0.22
	LVMass [g]	146.9 (±53.7)	121.7 to 172.0	0.36
	RVEDV [ml]	193.8 (±87.5)	150.3 to 237.3	0.09
	RVESV [ml]	94.8 (±48.3)	70.8 to 118.8	0.06
	RVSV [ml]	99.0 (±42.7)	77.7 to 120.3	0.64
	RVEF [%]	52.0 (±8.1)	48.0 to 56.0	0.11
**SSIR-nonBH**	LVEDV [ml]	197.2 (±81.2)	149.2 to 235.3	0.0096^*^
	LVESV [ml]	101.0 (±66.4)	70.0 to 132.1	0.57
	LVSV [ml]	96.2 (±22.4)	85.7 to 106.7	0.11
	LVEF [%]	52.5 (±12.6)	46.6 to 58.4	0.61
	LVMass [g]	143.5 (±52.0)	118.7 to 167.4	0.0495^*^
	RVEDV [ml]	193.4 (±85.6)	150.8 to 235.9	0.09
	RVESV [ml]	94.4 (±48.4)	70.3 to 118.5	0.06
	RVSV [ml]	98.9 (±41.3)	78.4 to 119.5	0.78
	RVEF [%]	52.2 (±9.3)	47.6 to 56.8	0.20

Note: SD: standard deviation, CI: confidence interval, vs. Ref: p-value from paired t-testing, LVEDV: left-ventricular end diastolic volume, LVESV: left-ventricular end systolic volume, LVEF: left-ventricular ejection fraction, LVMass: left-ventricular mass, LVSV: lef-ventricular stroke volume, RVEDV: right-ventricular end diastolic volume, RVESV: right-ventricular end systolic volume, RVSV: right-ventricular stroke volume, RVEF: right-ventricular ejection fraction.

**Table 2:** Linear regression and Bland-Altman analysis of (Ref) vs. study sequences with (SSIR-BH) and without (SSIR-nonBH) breath-hold.

		Linear regression analysis		Bland-Altman analysis	
		r^2^	slope	MD (±SD)	LOA
**Ref vs. SSIR-BH**	LVEDV [ml]	0.99	0.98	−2.7 (±0.9)	−4.4 −0.9
	LVESV [ml]	1.00	0.97	0.1 (±0.9)	−1.7 1.8
	LVSV [ml]	0.98	1.01	−2.7 (±0.7)	−4.1 −1.4
	LVEF [%]	0.98	0.97	−0.5 (±0.4)	−1.3 0.3
	LVMass [g]	0.97	1.07	−2.4 (±2.6)	−7.4 2.6
	RVEDV [ml]	1.00	0.95	2.8 (±1.6)	−0.2 5.9
	RVESV [ml]	0.99	0.95	2.4 (±1.2)	0.0 4.9
	RVSV [ml]	0.96	0.92	0.4 (±1.8)	−1.2 −2.0
	RVEF [%]	0.85	1.09	−0.7 (±0.1)	−0.9 −0.5
**Ref vs. SSIR-nonBH**	LVEDV [ml]	0.99	1.00	−3.1 (±1.2)	−5.4 −0.7
	LVESV [ml]	1.00	0.99	−0.6 (±1.0)	−2.4 1.3
	LVSV [ml]	0.93	1.05	−2.5 (±1.5)	−5.4 0.4
	LVEF [%]	0.96	0.98	−0.3 (±0.6)	−1.4 0.8
	LVMass [g]	0.96	1.10	−6.2 (±3.0)	−12.0 −0.4
	RVEDV [ml]	0.99	0.95	2.4 (±1.4)	−0.3 5.1
	RVESV [ml]	0.99	0.94	2.1 (±1.0)	0.1 4.2
	RVSV [ml]	0.92	0.93	0.3 (±1.2)	−11.0 −0.9
	RVEF [%]	0.84	0.97	−0.5 (±0.04)	−0.4 −0.6

Note: MD: mean difference, SD: standard deviation, LOA: limits of agreement, LVEDV: left-ventricular end diastolic volume, LVESV: left-ventricular end systolic volume, LVEF: left-ventricular ejection fraction, LVMass: left-ventricular mass, LVSV: left-ventricular stroke volume, RVEDV: right-ventricular end diastolic volume, RVESV: right-ventricular end systolic volume, RVSV: right-ventricular stroke volume, RVEF: right-ventricular ejection fraction.

**Table 3:** Intra- and inter-observer agreement statistics for volumetric and regional wall movement analysis for study sequences with (SSIR-BH) and without (SSIR-nonBH) breath-hold.

		LVEF	RVEF	RWM
**Intra-observer**	***SSIR-BH***	r^2^ = 0.97 slope = 1.08 p = 0.59	r^2^ = 0.95 slope = 0.90 p = 0.74	ĸ = 1
	***SSIR-nonBH***	r^2^ = 0.98 slope = 0.98 p = 0.49	r^2^ = 0.96 slope = 1.15 p = 0.78	ĸ = 0.98
**Inter-observer**	***SSIR-BH***	r^2^ = 0.95 slope = 0.98 p = 0.35	r^2^ = 0.83 slope = 0.70 p = 0.62	ĸ = 0.96
	***SSIR-nonBH***	r^2^ = 0.94 slope = 0.89 p = 0.33	r^2^ = 0.94 slope = 0.89 p = 0.71	ĸ = 0.94

**Note:** LVEF: left-ventricular ejection fraction, RVEF: right-ventricular ejection fraction, RMW: regional wall movement, r^2^ and slope values for LVEF and RVEF from linear regression analysis, p-value from paired t-testing, ĸ: Cohen’s kappa.

**Table 4:** Overview of sequence parameters used for the reference standard sequence (Ref) and the study sequence (SSIR).

	Ref	SSIR (BH/non-BH)
TR [msec]	3.3	2.8
TE [msec]	1.46	1.18
Slice thickness/gap [mm]	8/2	8/2
Inplane resolution [mm]	1.5 × 1.5	1.8 × 1.8
Flip angle [°]	43	32
Bandwidth [Hz/pixel]	970	900
Temporal resolution [msec]	43 (interpolated to 25 cardiac phases)	~40 (no interpolation)
Image Matrix	224 × 126	192 × 118
Acceleration factor	2 (GRAPPA)	11.3
Breath-holds	12–14	1/none
ECG mode	Retrospective gating	Prospective triggering
Acquisition time ± SD (Range) [sec]	392 ± 74 (279–552)	21 ± 4 (14–27)

**Note:** BH: breath hold, TR: repetition time, TE: echo time, GRAPPA: generalized autocalibrating partial parallel acquisition, SD: standard deviation.

## References

[b1] Pfeiffer M. P. & Biederman R. W. Cardiac MRI: A General Overview with Emphasis on Current Use and Indications. Med Clin North Am 99, 849–861, doi: 10.1016/j.mcna.2015.02.011 (2015). 26042886

[b2] Curtis J. P. . The association of left ventricular ejection fraction, mortality, and cause of death in stable outpatients with heart failure. J Am Coll Cardiol 42, 736–742 (2003). 12932612 10.1016/s0735-1097(03)00789-7

[b3] Fratz S. . Guidelines and protocols for cardiovascular magnetic resonance in children and adults with congenital heart disease: SCMR expert consensus group on congenital heart disease. J Cardiovasc Magn Reson 15, 51, doi: 10.1186/1532–429X-15–51 (2013). 23763839 PMC3686659

[b4] Kehr E., Sono M., Chugh S. S. & Jerosch-Herold M. Gadolinium-enhanced magnetic resonance imaging for detection and quantification of fibrosis in human myocardium *in vitro*. Int J Cardiovasc Imaging 24, 61–68, doi: 10.1007/s10554-007-9223-y (2008). 17429755

[b5] Doesch C. & Papavassiliu T. Diagnosis and management of ischemic cardiomyopathy: Role of cardiovascular magnetic resonance imaging. World J Cardiol 6, 1166–1174, doi: 10.4330/wjc.v6.i11.1166 (2014). 25429329 PMC4244614

[b6] Romero J., Xue X., Gonzalez W. & Garcia M. J. CMR imaging assessing viability in patients with chronic ventricular dysfunction due to coronary artery disease: a meta-analysis of prospective trials. JACC Cardiovasc Imaging 5, 494–508, doi: 10.1016/j.jcmg.2012.02.009 (2012). 22595157

[b7] Vincenti G. . Compressed sensing single-breath-hold CMR for fast quantification of LV function, volumes, and mass. JACC Cardiovasc Imaging 7, 882–892, doi: 10.1016/j.jcmg.2014.04.016 (2014). 25129517

[b8] Bogachkov A. . Right ventricular assessment at cardiac MRI: initial clinical experience utilizing an IS-SENSE reconstruction. Int J Cardiovasc Imaging, doi: 10.1007/s10554-016-0874-4 (2016). 27091733

[b9] Haubenreisser H. . Right Ventricular Imaging in 25 Seconds: Evaluating the Use of Sparse Sampling CINE With Iterative Reconstruction for Volumetric Analysis of the Right Ventricle. Invest Radiol, doi: 10.1097/RLI.0000000000000250 (2016). 26895192

[b10] Sudarski S. . Free-breathing Sparse Sampling Cine MR Imaging with Iterative Reconstruction for the Assessment of Left Ventricular Function and Mass at 3.0 T. Radiology. 151002, doi: 10.1148/radiol.2016151002 (2016). 27399326

[b11] Liu J. . Dynamic cardiac MRI reconstruction with weighted redundant Haar wavelets. Proc. Intl. Soc. Mag. Reson. Med. 20, 178 (2012).

[b12] Cerqueira M. D. . Standardized myocardial segmentation and nomenclature for tomographic imaging of the heart. A statement for healthcare professionals from the Cardiac Imaging Committee of the Council on Clinical Cardiology of the American Heart Association. Circulation 105, 539–542 (2002). 11815441 10.1161/hc0402.102975

